# Effect of sound therapy on whole scalp oscillatory brain activity and distress in chronic tinnitus patients

**DOI:** 10.3389/fnins.2023.1212558

**Published:** 2023-08-29

**Authors:** Mie Lærkegård Jørgensen, Petteri Hyvärinen, Sueli Caporali, Torsten Dau

**Affiliations:** ^1^Hearing Systems Section, Department of Health Technology, Technical University of Denmark, Kongens Lyngby, Denmark; ^2^WS Audiology, Lynge, Denmark; ^3^Copenhagen Hearing and Balance Center, Department of Oto-Rhino-Laryngology, Head and Neck Surgery and Audiology, Rigshospitalet, Copenhagen University Hospital, Copenhagen, Denmark; ^4^Department of lnformation and Communications Engineering, School of Electrical Engineering, Aalto University, Espoo, Finland

**Keywords:** sound therapy, tinnitus, oscillatory activity, treatment, gamma activity, longitudinal study

## Abstract

**Introduction:**

Sound therapy is a common tinnitus treatment, where the tinnitus percept is either fully or partially masked by an external sound. Some tinnitus patients experience a decrease in tinnitus related distress after the use of sound therapy. Differences in the neural response to sound therapy may form a basis for classifying tinnitus patients.

**Methods:**

In this study, the long-term (2 months) effects of sound therapy on the oscillatory brain activity and tinnitus related distress were investigated in chronic tinnitus patients. Baseline oscillatory activity in the group of tinnitus participants was also compared to a matched control group.

**Results:**

No differences were found in the oscillatory activity when comparing the tinnitus group to the control group. Differences were found for the frequency range between 27.5 and 41.5 Hz corresponding to high beta and gamma power when comparing the tinnitus group before and after the use of sound therapy. Furthermore, a reduction of the tinnitus-related distress was found after the long-term use of sound therapy. However, there was no correlation between the changes in the oscillatory activity and the reductions of the tinnitus-related distress.

**Discussion:**

Overall, the lack of correlation between the changes in tinnitus-related distress and changes in power activity hampers the interpretability of the findings and undermines the utility of using oscillatory activity as a biomarker for the effect of sound therapy treatment.

## 1. Introduction

Tinnitus is often perceived as a sizzling, hissing or ringing sound (Baguley et al., 2013) and has been defined as “the conscious awareness of a tonal or composite noise for which there is no identifiable corresponding external sound source” (De Ridder et al., 2021). The average prevalence of tinnitus in the European countries is 14.7%, ranging from 8.7% in Ireland to 28.3% in Bulgaria (Biswas et al., 2022). Severe tinnitus was found in 1.2% of the European population and was associated with cognitive deficits, increased stress, sleep difficulties (Crönlein et al., 2016), concentration difficulties, anxiety (Pattyn et al., 2016), depression (Salazar et al., 2019), and reduced quality of life (Nondahl et al., 2007; Weidt et al., 2016).

Several types of treatments are offered to tinnitus patients. These include, but are not limited to, hearing aids, stress reduction methods, tinnitus retraining therapy, masking devices, cognitive behavioral therapy and biofeedback therapy (Bhatt et al., 2016). Many of these treatments can be classified as sound therapy because they provide either amplification of the environmental sound scene, or present broadband noise, natural sounds, music, fractal tones or another type of sound to the patients. The intention of sound therapy is to diminish the tinnitus percept by either masking it completely so the tinnitus can no longer be heard during the application of sound therapy, or by masking the tinnitus partially, when the tinnitus may still be audible, but possibly perceived as less intrusive. Here, only the prevalence of sounds like noise and music, but not amplification, will be referred to as sound therapy. Sound therapy is noninvasive, simple and accepted by most participants which may explain why it is widely used in clinical practices. Despite sound therapy being widely available and the frequent use of the treatment for patients, evidence is still lacking regarding the effects of and mechanisms influenced by the sound therapy. Several systematic reviews have evaluated the effects of sound therapy. Although most studies have found a positive impact of the therapy on tinnitus, this evidence has been considered to be at a low level, mainly due to the lack of large randomized controlled trials (Phillips and McFerran, 2010; Hoare et al., 2011; Hobson et al., 2012; Sereda et al., 2018). Furthermore, the majority of studies have included additional treatments, such as counseling or amplification, when evaluating the effects of sound therapy (Sereda et al., 2018). Some of the studies that focused on the isolated effect of sound therapy found that the prolonged use of the therapy decreased the tinnitus related distress (Sweetow and Sabes, 2010; Tyler et al., 2020). Moreover, small but significant reductions of group-level average tinnitus related distress have been found after the use of sound therapy. However, while 30%–65% of the included participants experienced a reduction in the distress, the remaining participants did not experience any change or even experienced a worsening of the tinnitus-related distress (Sweetow and Sabes, 2010; Tyler et al., 2020). To further advance the understanding and personalization of sound therapy, it would be valuable to be able to divide patients into “responders” who benefit from the therapy and “non-responders” who do not benefit from the therapy and to understand the differences between the groups.

Several studies using either magneto- or electroencephalography (MEG, EEG) found altered oscillatory activity in tinnitus patients compared to control participants (Weisz et al., 2005, 2007b; Moazami-Goudarzi et al., 2010; Adjamian et al., 2012; Balkenhol et al., 2013; Schlee et al., 2014). Decreased activity in the alpha (8–12.5 Hz) power band and increased activity in the delta (2–3.5 Hz), and gamma (33–44 Hz) power bands have been reported (Weisz et al., 2005, 2007b; Moazami-Goudarzi et al., 2010; Adjamian et al., 2012; Balkenhol et al., 2013; Schlee et al., 2014). These alterations have been interpreted by Weisz et al. (2007a) in the framework of the “Synchronization-by-Loss-of-Inhibition-Model” that suggests that deprivation of auditory input causes loss of inhibition and a concomitant reduction of the alpha activity in the auditory cortex, while spontaneous synchronization enhances the gamma activity. Furthermore, Balkenhol et al. (2013) found that tinnitus related distress was associated with increased theta activity. The oscillatory activity may therefore represent a biomarker of tinnitus and might be used to instrumentally evaluate the effect of sound therapy treatments.

The present study investigated whether long-term sound therapy induces lasting changes in the EEG spectrum. First, the oscillatory brain activity between a group of chronic tinnitus participants and a control group without tinnitus was compared. Then, to evaluate possible changes in the oscillatory activity caused by the sound therapy, the oscillatory activity before and after the use of sound therapy was compared. Finally, it was investigated whether the change in oscillatory activity with sound therapy was related to the tinnitus related distress and the power bands of the resting state EEG. Based on previous findings (Weisz et al., 2005, 2007b; Adjamian et al., 2012; Balkenhol et al., 2013; Schlee et al., 2014), it was hypothesized that the tinnitus group would show reduced activity in the alpha band and increased activity in the delta, gamma and theta bands compared to the control group. Furthermore, it was hypothesized that the sound therapy would revert these patterns and thus increase the alpha activity and decrease the delta, gamma and theta activity compared to the baseline activity in those tinnitus participants who respond to the treatment.

## 2. Materials and methods

### 2.1. Participants

**Figure 1:**
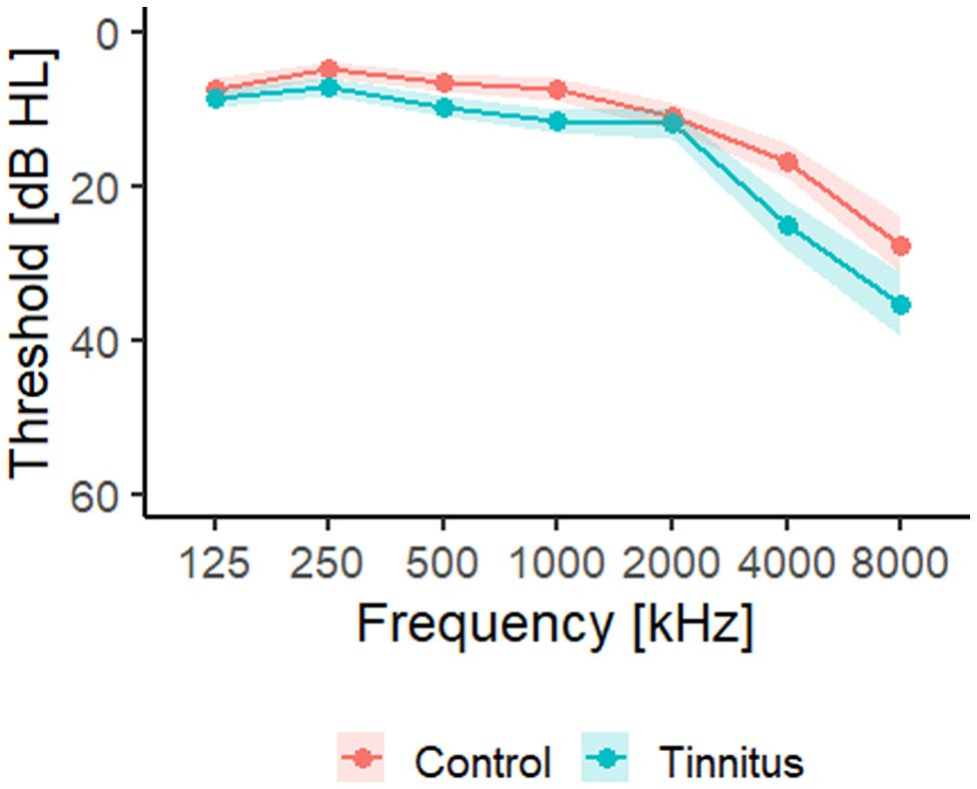
Average audiogram. Hearing thresholds were measured from 125 Hz to 8 kHz. Results were averaged for the left and right ear and shown with mean ± SEM. The red line is the average for the control group, while the blue line is the average threshold for the tinnitus group. Adapted from Joergensen et al. (2022).

Twenty chronic tinnitus participants (14 men, 6 women) aged between 23 and 72 years (51.7 ± 14.2 years) and with an average Pure Tone Average (PTA) of 14.6 ± 12.3 dB HL (mean ± SD) were included in the study. Figure 1 shows the average hearing thresholds of the tinnitus and control groups. Prior to inclusion in the study, the participants were asked to fill in the Tinnitus Handicap Inventory (THI) questionnaire and only participants experiencing a negative tinnitus reaction (defined as a THI score above 18) were included in the study. The majority of the participants (14 of the 20) described their tinnitus percept as a tone, while three participants described the percept as noise-like. One participant perceived two concurrent tinnitus percepts and two participants were unsure about whether the percept was tonal or noisy. The tinnitus pitch was described as either a high-frequency or a very high-frequency percept by 18 participants. One participant could not identify the tinnitus pitch and one participant perceived two tinnitus percepts: one was identified as a high-frequency sound while the other was identified as a low-frequency sound. 14 participants described that they could mask their tinnitus perception with external sounds (e.g., TV or radio), two participants could not mask their percept, while the remaining four participants did not know if their tinnitus could be masked or not. A detailed overview of the participant’s tinnitus perception can be found in Table 1. Twenty participants who reported no history of chronic tinnitus and who experienced spontaneous tinnitus less than twice a month were included as a control group. The participants were matched with regards to age, gender and hearing loss (average PTA was 10.5 ± 10.3 dB HL). The participants of this study were the same as those included in Joergensen et al. (2022), where the effect of sound therapy on visual attention was investigated in chronic tinnitus patients.

**Table 1:** Description of tinnitus percept based on answers from the Tinnitus Sample Case History Questionnaire.

Participant	Age	Gender	Tinnitus duration (years)	Tinnitus location	Tinnitus loudness	Tinnitus percept	Tinnitus pitch	Tinnitus masking	Pre-THI	Post-THI	Tinnitus etiology
1	64	M	6	BE	100	Tone	VHF	Yes	60	62	Head injury
2	68	M	10	IH	70	Tone	HF	No	58	32	Unknown
3	54	F	11	BE (L)/IH	65	Noise	Do not know	Yes	38	16	Change in hearing
4	23	M	10	IH	40	Tone	VHF	Yes	44	28	Very loud sound
5	72	F	20	LE	75	Noise	HF	Do not know	64	76	Very loud sound
6	24	M	8	BE (R)	15	Tone/Noise	VHF	Yes	44	42	After a cold
7	54	M	10	LE/BE (L)	20	Tone	HF	Yes	18	10	Unknown
8	32	M	5	BE/IH	70	Tone	VHF	No	62	58	Stress
9	39	M	10	BE	75	Tone	VHF	Yes	30	28	Stress
10	57	M	24	BE	57.5	Tone	VHF/HF	Yes	48	16	Very loud sound
11	58	M	12	BE	30	Noise	HF	Yes	22	16	Very loud sound
12*	60	F	3	RE	70	Tone/noise	HF/LF	Yes	46	32	Head injury
13	46	M	>20	IH	85	Tone	VHF	Yes	54	38	Very loud sound
14	68	M	30–40	BE (R)	70	Tone	HF	Do not know	14^**^	12	Unknown
15	52	F	5–6	IH	65	Noise/tone	HF	Yes	24	24	Unknown
16	61	M	15	RE	60	Tone	HF	Yes	18	18	Meniéres^***^
17	46	M	28	IH	60	Tone	HF	Yes	34	30	Very loud sound
18	37	M	1	LE	50	Tone	HF	Yes	40	20	Very loud sound
19	69	F	20	BE (R) /IH	75	Tone	HF	Do not know	44	32	Change in hearing
20	50	F	33	BE	80	Tone	VHF	Do not know	24	12	Very loud sound

Table adapted from Joergensen et al. (2022). ^*^Perceives two different sounds. ^**^On day of prescreening the THI-score was >18 and participant was therefore included in study. However, during first session a THI score of 14 was measured. ^***^No Meniéres episodes had been experienced in the past 1.5 years. The tinnitus etiology is self-reported. Gender: M, male; F, female. Tinnitus location: BE, Both ears equally; BE(R), Both ears, mainly the right ear; BE(L), Both ears, mainly the left ear; LE, Left ear; RE, Right ear; IH, Inside head. Tinnitus pitch: VHF, Very High-frequency; HF, High-frequency; MF, Medium frequency; LF, Low-frequency.

All participants were recruited from either the local volunteer database at the Hearing Systems Section at the Technical University of Denmark (DTU), from a dedicated trial website^1^ or from the local volunteer database at WS Audiology. The study took place at the Hearing Systems Section at the Department of Health Technology, DTU. The study was approved by the Science-Ethics Committee for the Capital Region of Denmark (reference H-16036391). All subjects provided written informed consent before that start of the study.

### 2.2. Audiometry

Pure-tone audiometry was conducted in a sound-proof booth using a standard clinical audiometer (model AS216, Interacoustics A/S, Middlefart, Denmark) and HD200 headphones (Sennheiser GmbH & Co. KG, Wedermark, Germany) in the frequency range from 125 Hz to 8 kHz.

### 2.3. Questionnaires

Tinnitus severity was evaluated using the standardized outcome questionnaire, the Tinnitus Handicap Inventory (THI; Newman et al., 1996). The THI contains 25 questions that can be answered with “yes” (4 points), “sometimes” (2 points), and “no” (0 points), resulting in an overall THI score between 0 and 100 points. Furthermore, patient case history was collected with the Tinnitus Sample Case History Questionnaire (TSCHQ; Langguth et al., 2007). The TSCHQ gathers descriptive information about the participants’ tinnitus including tinnitus perception, pitch and loudness.

### 2.4. EEG acquisition

EEG was recorded in two sessions: (1) a pre-treatment baseline measure and (2) a post-treatment measure after 2 months of sound therapy treatment for the tinnitus group. The recordings took place in an electrically shielded soundproof booth and the participants were seated in an armchair. Four minutes of resting-state continuous EEG were recorded in awake state and the participants were asked to keep their eyes closed throughout the recording. The Biosemi ActiveTwo system was used with 64 active Ag/AgCl electrodes and the electrodes were placed in a Biosemi 64 electrode head cap according to the standard international 10–20 system. The Common Mode Sense (CMS) electrode was used as a reference and placed over C1 whereas the Driven Right Leg (DRL) electrode was used as ground and placed over C2. SignaGel electrode gel was applied to increase conductivity. The EEG signals were recorded at a sampling rate of 2,048 Hz and the recordings were pre-processed off-line.

### 2.5. EEG data pre-processing and analysis

The EEG was pre-processed using the MATLAB (R2021b) and EEGLAB software (2021.1, Delorme and Makeig, 2004). The EEG data were first down-sampled to 256 Hz and then re-referenced to an average reference of all electrodes. Then the data were filtered with a 0.5 Hz high-pass finite impulse response filter and bad channels were visually identified and replaced using interpolation of nearby channels. Independent Component Analysis (ICA) was used to remove ocular and muscular artifacts. The EEG was divided into 2 s epochs and manually inspected for artifacts.

To conduct the power spectrums, the raw data was first windowed with a Hanning taper and then a Fast-Fourier-Transform was performed in the frequency range from 0.25 to 45 Hz. The power spectrums were then normalized for each participant with the mean power between 2 and 44 Hz.

The between-participant contrast used to compare the tinnitus and control participants at baseline was conducted with a cluster-level permutation test with the ft_freqstatistics in FieldTrip (Oostenveld et al. 2011). For the permutation test, the Monte Carlo method and an independent samples T-statistic was used to evaluate the effect. Furthermore, the critical alpha level was chosen to 0.05 and a two-sided statistical test with 10.000 random permutations was conducted. Similarly, the within-participant contrast used to compare the tinnitus participants before and after the use of sound therapy was also conducted with a cluster-level permutation test with the ft_freqstatistics in FieldTrip. For the permutation test, the Monte Carlo method and a dependent samples T-statistic was used to evaluate the effect. Furthermore, the critical alpha level was chosen to 0.05 and a two-sided statistical test with 10.000 random permutations was conducted.

The statistically significant clusters will be plotted with the ft_clusterplot function. The topoplots show the statistical results of the cluster-based permutation test (T-statistics). The cluster statistics presented in the result section represent the sum of sample-specific T-statistics belonging to the specific cluster. The largest cluster-level statistics of the different clusters is chosen as the actual test statistic.

The relationship between the EEG data and changes in THI scores, likeness of the sound therapy and use of the sound therapy were tested with a permutation test. The ft_statfun_correlationT function in FieldTrip was used for the tests. A Spearman correlation test with a alpha level of 0.05 was conducted.

### 2.6. Sound therapy

All participants were fitted bilaterally with Widex Evoke Passion 440 hearing aids (HA) with instant open ear tips. All fittings were performed using the Widex fitting software—Compass GPS (v. 3.4). To avoid the possible effect of amplification as tinnitus treatment, the participants were not compensated for their hearing losses. Instead, all participants were fitted based on a flat audiogram with hearing thresholds of 10 dB at all frequencies from 125 Hz to 8 kHz.

Fractal tones were used as sound therapy in the present study. Fractal tones are harmonic and melodic tones that are unpredictable, but without any sudden changes in tonality and tempo (Tyler et al., 2017). The fractal tones are randomly generated by a patented algorithm in the HA (patent no. DK/EP 2559263, 20191211). A random number sequence is generated by the fractal generator. These numbers are assigned their pitch, tempo, and intensity based on predefined rules. In the current study, the standard parameters from the Compass GPS fitting software were used for all participants. 12 music generators decode these numbers and generate the desired tones. Moreover, the melodic structures are based on a pseudorandom signal, generated by a maximum length sequence generator. The HA were programmed with a custom-made MATLAB script to ensure that fractal tone program was the only one available the HA. The participants evaluated the pleasantness of the individual fractal tones and choose the one they preferred. The participants could choose between 5 fractal tone types (“aqua,” “coral,” “green,” “lavender,” and “sand”). The preferred fractal tone was programmed into the HA. The average pleasantness for the tinnitus participants were 8.0 ± 1.2 points (mean ± standard deviation on a scale from 0 to 10). The participants were guided to make sure that the fractal tones were audible when they were used, but that they should not interfere with conversational speech. All of the participants were given a remote control to adjust the level of the fractal tones to ensure that the therapy could be used comfortably in different environments. The participants were asked to use the sound therapy for at least 3–4 h per day. Datalog data from the HA showed that the HA had on average been active for 6.0 ± 4.4 h per day (mean ± standard deviation, data from 18 participants due to missing data: Two participants lost their hearing aids during the study. These HA were immediately replaced with new ones, but the datalogs could not be retrieved from the lost HA).

### 2.7. Procedure

All tinnitus participants were selected based on a hearing screening that assessed both the participants’ audiogram and their tinnitus perception. Prior to inclusion in the study, the participants were asked to fill out the tinnitus handicap inventory (THI) questionnaire and only participants experiencing a negative tinnitus reaction (defined as a THI score above 18) were included in the study. The matched control group participants without experienced tinnitus were selected from the Hearing Systems database based on their gender, age and hearing level.

In the first session, all participants were given a thorough introduction to the aim of the study and the included methods before they were asked to provide written informed consent. HAs were then fitted with the GPS fitting software and a customized Matlab script. The customized Matlab script was used to remove the standard universal program of the HAs to make sure that the participants could only use the HAs to play the sound therapy. Each fractal tone program was evaluated by the participants who then selected the most pleasant fractal tone. This sound was then used for the remaining part of the study.

The participants wore an EEG electrode cap where electrodes were attached according to the standard international 10–20 system. Four minutes of resting-state EEG was recorded while the participants were seated in an armchair in a darkly lit soundproof booth and were instructed to keep their eyes closed during the recording.

The tinnitus participants were then asked to use the sound therapy on a daily basis for at least 3–4 h per day. After 2 months of sound therapy use, the tinnitus participants took part in a second session. In the second session, the participants completed the THI questionnaire before the resting state EEG recordings were carried out as described above for the first session. The second session was planned to take place at the same time of the day (±2 h) as in the first session to limit any possible effects of the circadian rhythms on the resting state EEG.

### 2.8. Behavioral statistical analysis

The statistical analyses were performed with the R statistical software (R version 4.0.3, R Foundation for Statistical Analysis, Austria) with the packages “tidyverse” and “ggpubr.” A paired *t*-test was used to compare the pre and post treatment THI scores.

## 3. Results

### 3.1. Behavioral assessment

**Figure 2:**
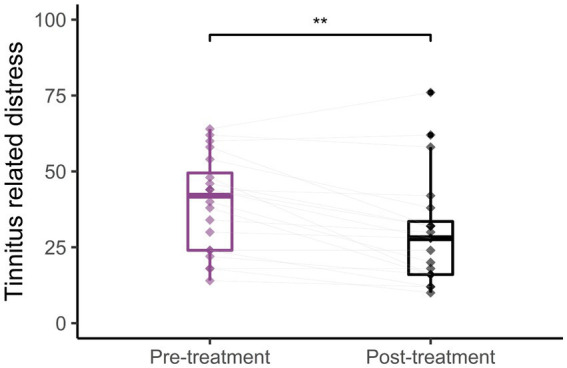
Tinnitus distress. Average THI scores before (left) and after (right) long-term treatment with sound therapy (^**^p < 0.01). Adapted from Joergensen et al. (2022).

Figure 2 shows the average change of the THI scores from the pre to the post sound therapy treatment. The average pre-treatment THI score was 39.3 ± 3.5 points, which is categorized as a moderate tinnitus handicap, while the average post-treatment THI score was 30.1 ± 4.0 points which is categorized as a mild tinnitus handicap. There was a statistically significant difference between the average pre-treatment THI scores and the post-treatment THI scores [*t*(19) = 3.84, *p* = 0.0011]. The average change of the THI score after the use of sound therapy was 9.2 ± 2.4 points. 16 participants experienced a decrease in the THI score, two participants did not show changes in the THI score and two participants experienced an increase of the THI score after the treatment. Of the 16 participants experiencing a reduction of the THI score, 9 experienced clinically significant reductions, defined as an improvement of more than 7 points as suggested by Zeman et al. (2011).

### 3.2. EEG power band assessment

#### 3.2.1. Control vs. tinnitus

**Figure 3:**
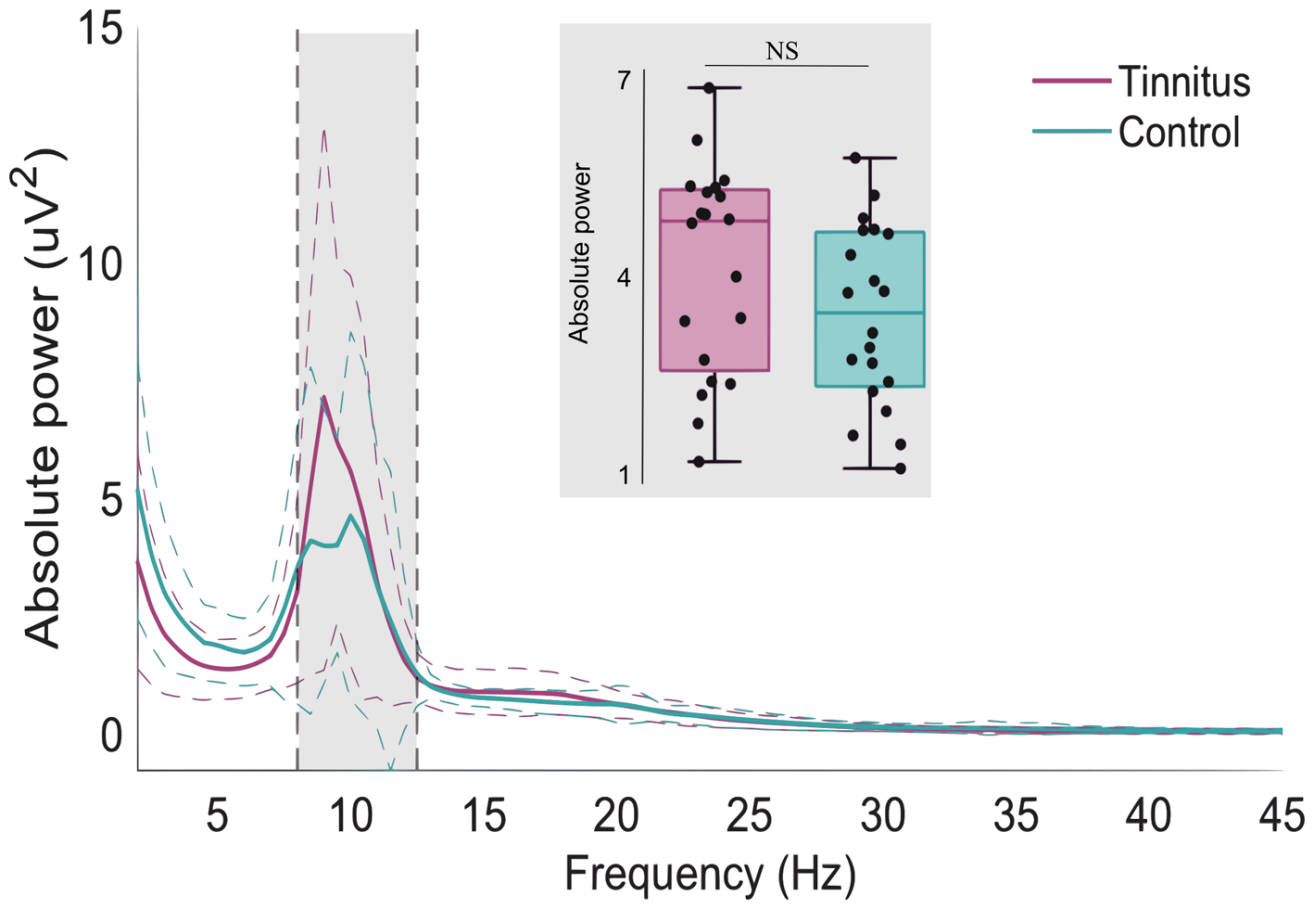
Whole scalp normalized power spectra for the tinnitus group (purple) and the control group (green) for the frequencies between 1 and 45 Hz. The dashed lines represent the standard deviation of the participants in the whole scalp normalized power. The alpha frequency range (8–12.5 Hz) is marked in gray. The gray insert shows the box plots and individual alpha activity (black dots) averaged across the whole scalp.

The normalized oscillatory activity was compared between the tinnitus group and the matched control group for the whole spectrum. Figure 3 shows the normalized power activity for the tinnitus group (purple line) and the control group (blue line). No statistically significant differences were found between the two groups (Cluster statistic = −149.1, value of *p* > 0.05). The alpha frequency range (8–12.5 Hz) is highlighted with gray in Figure 3 and shows a trend toward a higher alpha power activity in the tinnitus group than in the control group, but there was no statistically significant difference. The insert (gray box) shows the box plots of the alpha activity and the spread in the individual alpha power activity averaged across all scalp electrodes.

#### 3.2.2. Pre-treatment vs. post-treatment

**Figure 4:**
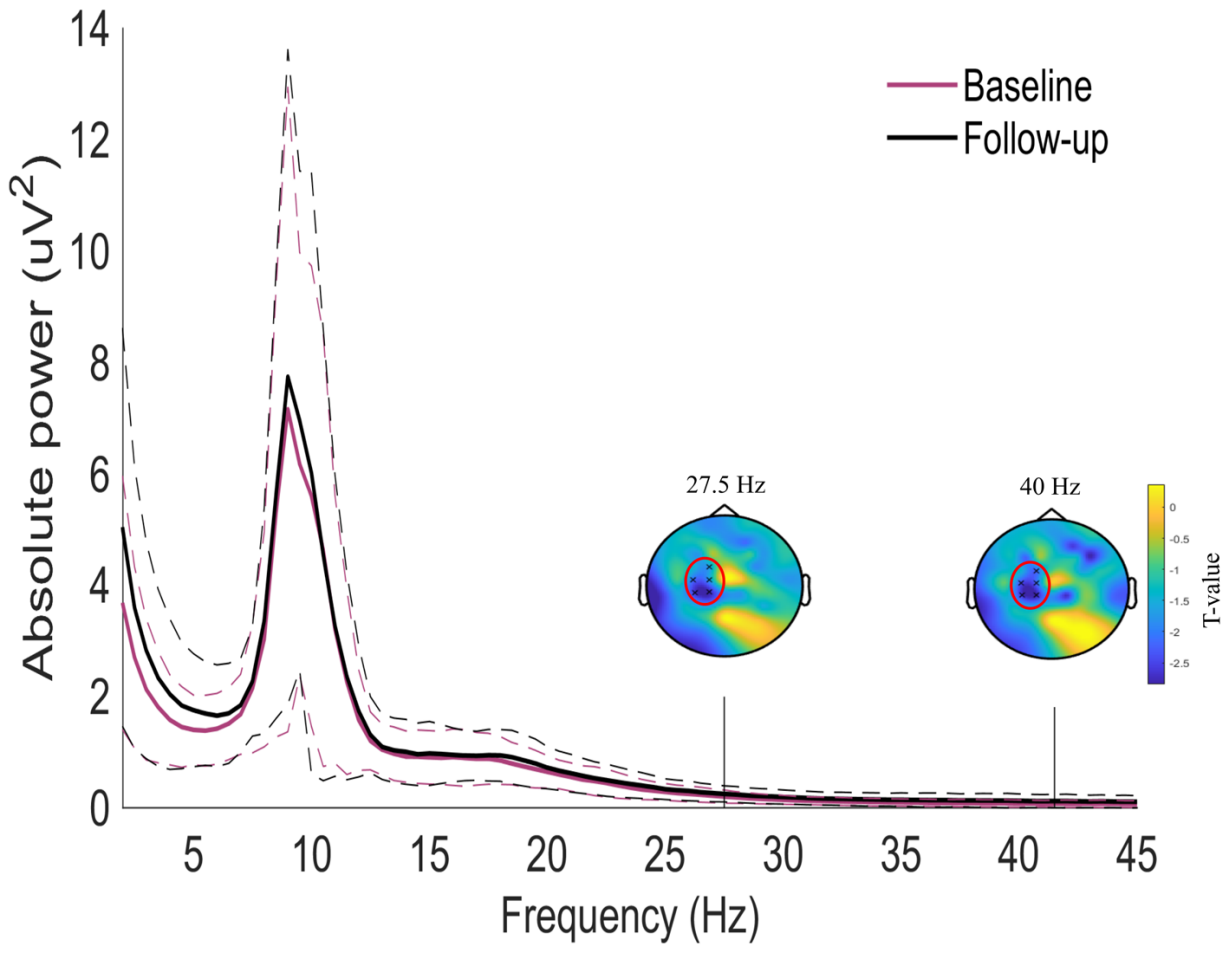
Whole-scalp normalized power spectra for the tinnitus group before (purple) and after (black) sound therapy treatment in the frequency range between 1 and 45 Hz. The dashed lines represent the standard deviation of the participants in the whole scalp normalized power. A significant cluster was found between 27.5 and 41.5 Hz. The two topoplots show the statistical results (red circles) of the cluster.

Figure 4 shows the normalized difference in oscillatory activity between the pre- and post-treatment conditions for the tinnitus participants. A statistically significant cluster was found in the frequency range from 27.5 to 41.5 Hz corresponding to the high beta and the gamma frequency ranges (Cluster statistic = −132, value of *p* = 0.012). The cluster is indicated in the red circles on the topographic plots of the cluster statistic results (*t*-values). This indicates that the tinnitus participants had increased power in the frequency range from 27.5 to 41.5 Hz after long-term use of sound therapy.

#### 3.2.3. Correlation between EEG and tinnitus

The statistically significant cluster found in the EEG power change from pre to post sound therapy treatment was compared with the change in tinnitus distress from pre to post treatment. No correlation was found between the power spectra changes within the cluster and the change in THI. Furthermore, there were no correlations between the power spectral changes, the likeness of the selected sound therapy tone and the datalog data from the HAs that estimated the amount of time the participants had used their HAs on a daily basis.

## 4. Discussion

The present study investigated whether long-term sound therapy induced changes in the EEG spectrum of tinnitus patients. The results showed a reduction in the tinnitus related distress after long-term sound therapy treatment. No differences were found in the oscillatory activity when comparing the tinnitus group with the matched control group without tinnitus. After the long-term sound therapy treatment, an increase in the gamma power was found for the tinnitus group.

Consistent with earlier findings (Sweetow and Sabes, 2010b; Melanie Herzfeld et al., 2014; Tyler et al., 2020), the present study showed a significant decrease of the tinnitus related distress after long-term use of sound therapy. 45% of the participants experienced a clinical reduction in the distress, 35% experienced a minor non-clinical reduction in the distress, 10% experienced no change in their distress and 10% experienced a worsening in the distress. Based on the “Synchronization-by-Loss-of-Inhibition-Model” (Weisz et al., 2007a), it was hypothesized that the tinnitus group would show decreased alpha power and increased delta and gamma power when compared to the control group. In contrast to the hypothesis and previous findings (Weisz et al., 2007b; Moazami-Goudarzi et al., 2010; Adjamian et al., 2012; Adamchic et al., 2014), the present study found no differences in the alpha, delta and gamma activity in the tinnitus group when compared to the control group. Moreover, the data showed a trend toward a higher level of alpha activity in the tinnitus participants than in the control participants. Differences in the study design may explain the discrepancies between the findings. In the present study, the hearing losses of the participants in the tinnitus and control groups were matched. This was not the case in most of the previous studies (Weisz et al., 2005, 2007b; Moazami-Goudarzi et al., 2010; Adamchic et al., 2014; Schlee et al., 2014). Furthermore, unlike previous studies, the resting-state EEG was only measured with closed eyes and not with alternating closed and open eyes as in several previous studies that investigated the difference between tinnitus and control participants (Moazami-Goudarzi et al., 2010; Adjamian et al., 2012). It has been shown that EEG measures with open eyes result in topographic changes in the power bands, whereby one study reported a general reduction in activity across all frequency bands (Barry and De Blasio, 2017) whereas another study showed alterations in the delta, theta and beta bands but not in the alpha band (Barry et al., 2007). The results reported in the present study can thus not directly be compared to the previous findings. However, the previous findings on oscillatory brain activity were not univocal and some studies reported reduced delta activity (Hébert et al., 2011; Pawlak-Osińska et al., 2013) in tinnitus patients when compared to control groups. Furthermore, similar to the findings from the present study, several other studies did not find a decreased alpha activity in tinnitus patients (Ashton et al., 2007; Lorenz et al., 2009; Moazami-Goudarzi et al., 2010; Vanneste et al., 2010; Hébert et al., 2011; Ortmann et al., 2011; Adjamian et al., 2012; Tass et al., 2012; Meyer et al., 2014).

Weisz et al. (2007a) suggested that gamma activity is related to tinnitus perception such that a reduced tinnitus percept would correspond to a decrease in gamma activity. In contrast to this proposal, the present study observed an increase in gamma activity for tinnitus participants after long-term use of sound therapy. This finding is consistent with results from previous studies that reported increased gamma activity during residual inhibition after short-term acoustic stimulation (Sedley et al., 2012, 2015; King et al., 2020; Schoisswohl et al., 2021). However, the findings are not directly comparable since the present study investigated the oscillatory changes after long-term use of sound therapy and because the participants did not listen to the sound therapy immediately prior to the EEG measurement. Previous studies have investigated the changes in oscillatory activity in chronic tinnitus patients after the use of an acoustic treatment, however the findings are not consistent with the findings in the present study. Music combined with Cognitive-Behavioral Therapy increased the power in the alpha and theta bands, while Acoustic Coordinated Reset neuromodulation decreased the gamma power and increased the alpha power (Adamchic et al., 2014; Feng et al., 2020). The lack of a correlation between the oscillatory activity and the reduction in the tinnitus related distress hampers the interpretability of changes in the gamma activity as a relevant biomarker of tinnitus related distress.

In the present study, no tinnitus control group was included to compare the effects of the sound therapy treatment with another treatment or a waiting list condition and the control group without tinnitus was only measured at baseline. A tinnitus control group was not included because the purpose of the present study was not to evaluate the effect of the sound therapy as a tinnitus treatment. The purpose was to investigate if the effect of the sound therapy affected the oscillatory activity in tinnitus patients. However, since no correlation was found between the changes in tinnitus distress and changes in the oscillatory activity, it would have been valuable to investigate if a corresponding decrease in gamma activity would be found in a control group without tinnitus after long-term use of sound therapy. The inability to conclude if the changes in gamma activity are related to the tinnitus percept or if similar changes would be found in control participants after long-term use of sound therapy is a limitation of the present study. Furthermore, no psychoacoustic measurements of the tinnitus loudness were included in the present study and no follow-up questionnaire about the tinnitus loudness was included. Previous studies found relationships between both alpha and gamma activity with tinnitus loudness (van der Loo et al., 2009; Balkenhol et al., 2013; Müller et al., 2013; King et al., 2020). It is therefore possible that the differences found in the oscillatory activity are correlated with the tinnitus loudness but not with the tinnitus related distress and future studies would benefit from including additional outcome measures.

Overall, although the present study found differences in the oscillatory activity after long-term use of sound therapy, the changes were not correlated with the improvements in the tinnitus related distress that the participants experienced. A better understanding of the sound therapy induced changes in the oscillatory activity and their relation to the tinnitus percept is needed before the oscillatory activity can be considered as a biomarker. Furthermore, the possible differences in brain activity between tinnitus patients and controls need to be further explored. This can involve various approaches, including but not limited to source-based or connectivity analyses. In addition, the results also showed large individual differences in the effects of the treatment on the tinnitus related distress, emphasizing the need for further improvement and individualization of the treatment.

## Data availability statement

The raw data supporting the conclusions of this article will be made available by the authors, without undue reservation.

## Ethics statement

The studies involving human participants were reviewed and approved by the Science-Ethics Committee for the Capital Region of Denmark (reference H-10916036391). The patients/participants provided their written informed consent to participate in this study. Written informed consent was obtained from the individual(s) for the publication of any potentially identifiable images or data included in this article.

## Author contributions

MJ, PH, SC, and TD contributed to the study conception and design. Study setup and analysis were performed by MJ and PH. Data collection was performed and first draft of the manuscript was written by MJ. Supervision was given by PH, SC, and TD. All authors contributed to the article and approved the submitted version.

## Funding

This work was supported by the European Research Council under the European Union’s Horizon 2020 research and innovation program (grant agreement no. 76604). The funders had no role in study design, data collection and analysis, decision to publish, or preparation of the manuscript.

## Conflict of interest

MJ and SC were employed by WS Audiology.

The remaining authors declare that the research was conducted in the absence of any commercial or financial relationships that could be construed as a potential conflict of interest.

## Publisher’s note

All claims expressed in this article are solely those of the authors and do not necessarily represent those of their affiliated organizations, or those of the publisher, the editors and the reviewers. Any product that may be evaluated in this article, or claim that may be made by its manufacturer, is not guaranteed or endorsed by the publisher.

## Footnotes

^1^www.forsoegsperson.dk
